# A prediction model of pulmonary hypertension in preterm infants with bronchopulmonary dysplasia

**DOI:** 10.3389/fped.2022.925312

**Published:** 2022-07-22

**Authors:** Chenhong Wang, Xiaolu Ma, Yanping Xu, Zheng Chen, Liping Shi, Lizhong Du

**Affiliations:** ^1^Neonatal Intensive Care Unit, Children’s Hospital, Zhejiang University School of Medicine, Hangzhou, China; ^2^National Clinical Research Center for Child Health, National Children’s Regional Medical Center, Hangzhou, China

**Keywords:** preterm infant, bronchopulmonary dysplasia, pulmonary hypertension, prediction model, nomogram

## Abstract

**Objective:**

Pulmonary hypertension (PH) is a severe cardiovascular complication of bronchopulmonary dysplasia (BPD) that contributes to the high mortality rates for preterm infants. The objective of this study is to establish a prediction model of BPD-associated PH (BPD-PH) by integrating multiple predictive factors for infants with BPD.

**Method:**

A retrospective investigation of the perinatal clinical records and data of echocardiography in all the preterm infants with BPD was performed from January 2012 to December 2019. A prediction model of BPD-PH was established based on the univariate and multivariate logistic regression analysis of the clinical data and evaluated by using the area under the receiver operating characteristic (ROC) curve (AUC), combined with the Hosmer–Lemeshow (HL) test. Internal validation was performed with bootstrap resampling.

**Result:**

A total of 268 infants with BPD were divided into the BPD-PH group and the no-PH group. Multivariate logistic regression analysis showed that the independent predictive factors of BPD-PH were moderate to severe BPD, small for gestational age, duration of hemodynamically significant patent ductus arteriosus ≥ 28 days, and early PH. A prediction model was established based on the β coefficients of the four predictors. The area under the ROC curve of the prediction model was 0.930. The Hosmer–Lemeshow test (*p* = 0.976) and the calibration curve showed good calibration.

**Conclusion:**

The prediction model based on the four risk factors predicts the development of BPD-PH with high sensitivity and specificity and might help clinicians to make individualized interventions to minimize the disease risk.

## Introduction

During the past three decades, since great progress in perinatal-neonatal medicine has been made in China, the survival of extremely premature infants has markedly increased. However, the developing lungs are susceptible to various injuries, which cause a rising incidence of bronchopulmonary dysplasia (BPD) (1). In China, based on previously published studies, the incidence of BPD in extremely premature infants with a gestational age of less than 28 weeks has increased from 19.3% in 2011 to 51.7% in 2019 (2, 3).

Survivors with BPD are at risk of cardiovascular complications because of the disruption of vascular growth and signaling (4, 5). Pulmonary hypertension (PH) is common in infants with moderate or severe BPD, with the reported incidence ranging from 8 to 38% (6–9). PH contributes significantly to high mortality and is associated with dependency on respiratory support, prolonged hospitalization, impaired neurodevelopment, and significant medical costs in infants with BPD (10, 11). In our previously published data, the mortality of BPD infants complicated with PH was 40.5% (12).

The pathogenesis of BPD-associated PH (BPD-PH) is complicated, including multiple factors, such as perinatal stress, abnormal lung development, and postnatal conditions. Therefore, identification of the risk factors is important for early prevention. However, a single predictive factor is difficult to apply in clinical practice due to its inconsistent standards, low sensitivity, and specificity. A prediction model developed by integrating multiple predictive factors may improve the accuracy of prediction. To test the hypothesis, we performed this retrospective study.

## Materials and methods

### Study population and data collection

Preterm infants admitted to the Level III neonatal intensive care unit (NICU) at Children’s Hospital of Zhejiang University School of Medicine between January 2012 and December 2019 who were diagnosed with BPD were eligible for this study. We excluded infants with congenital heart diseases [except patent ductus arteriosus (PDA), atrial septal defect, or patent foramen ovale], other major congenital anomalies, incomplete data, or death before 36 weeks’ postmenstrual age (PMA). This study was approved by the Ethics Committees of our hospital with a waiver of parental consent.

Clinical data were collected retrospectively from the electronic NICU database. Prenatal data included maternal demographics, oligohydramnios (defined as amniotic fluid index ≤ 5), prolonged rupture of membrane, pregnancy-related complications, antenatal corticosteroids use, and mode of delivery. Neonatal data included gestational age (GA), birth weight (BW), sex, the Apgar score at 1 and 5 min, small for gestational age (SGA) status, cumulative days of invasive mechanical ventilation (IMV) before 36 weeks’ PMA, and co-morbidity about respiratory distress syndrome (RDS) with surfactant use, hemodynamically significant PDA (hsPDA), pneumonia with positive respiratory culture, culture proven sepsis, PDA ligation, severe intraventricular hemorrhage (IVH), and necrotizing enterocolitis (NEC).

### Definitions

Pulmonary hypertension was evaluated non-invasively with echocardiography by a board-certified pediatric sonographer in cardiology and diagnosed based on either of the following criteria (7, 13, 14), including the estimated systolic pulmonary arterial pressure (sPAP) > 50% of the systemic arterial pressure, end-systolic flattened or left-deviated of the interventricular septum (IVS) with or without right ventricular dilatation, bidirectional or right-to-left shunt at the patent foramen ovale, or ductus arteriosus. When there was a tricuspid regurgitation (TR), sPAP was calculated by adding right atrial pressure (5 mmHg) to the right ventricle pressure gradient, which was estimated by the Bernoulli equation. In the absence of a measurable TR, we relied upon a PDA/patent foramen ovale gradient to estimate sPAP. Early PH was defined as having evidence of PH based on the assessment of echocardiography between 72 h and 14 days (15), while BPD-PH was defined as having evidence of PH beyond 36 weeks’ PMA in preterm infants with BPD (7).

Bronchopulmonary dysplasia was diagnosed based on the 2019 criteria proposed by Jensen (16). BPD severity was categorized according to the following modes of respiratory support administered at 36 weeks PMA, grade 1, nasal cannula at flow rates ≤ 2 L/min; grade 2, nasal cannula at flow rates > 2 L/min or non-invasive positive airway pressure; and grade 3, IMV.

Hemodynamically significant PDA was defined as the PDA diameter exceeding 1.5 mm combined with diastolic flow reversal in the descending aorta and the ratio of the left atrium to aorta greater than 1.5, as well as the clinical findings of pulmonary overcirculation, left heart overload, and/or poor systemic perfusion (17). SGA was defined as a birth weight less than the 10th percentile for gestational age (18). NEC was defined as modified Bells’ stage II criteria or greater (19). Pneumonia includes both congenital and hospital acquired pneumonia, which is defined as having chest X-ray findings with positive tracheal aspirate culture. Sepsis was defined as infants with a positive blood culture who were treated with antibiotics for at least 5 days. Severe IVH was defined as Papile’s Grade III or greater (20).

### Echocardiography screening protocol for hemodynamically significant PDA and pulmonary hypertension

All the preterm infants who were admitted before 2 weeks of life were evaluated by an echocardiogram 2–3 times a week for the hemodynamic status of PDA, pulmonary hypertension, and cardiac function. After 2 weeks of age, the frequency of echocardiogram was changed to 1–2 times a week. For all the preterm infants who developed increasing oxygen or respiratory support requirements in the late stage of life (usually after 4 weeks of life), PH was screened by an echocardiogram. When PH was confirmed, a series of echocardiograms were repeated every 1 or 2 weeks to monitor the dynamic change of PH and the response to interventions. If PH resolved, the echocardiogram was repeated monthly until weaning off respiratory support for 6 months.

### Statistical analyses

Data were presented as mean ± standard deviation (SD), median [interquartile range (IQR)], or number (percentage) where appropriate. Univariate analyses were performed using an independent *t*-test or non-parametric tests for continuous variables, and the Chi-square or Fisher’s exact test for categorical variables. Multivariate logistic regression was performed to identify the risk factors associated with BPD-PH. A prediction model and nomogram were established based on the multivariate logistic regression analysis of BPD-PH.

The area under the receiver operating characteristic (ROC) curve (AUC) was measured to assess the discriminative performance of the prediction model. A calibration curve was generated for the evaluation of calibration, combined with the Hosmer–Lemeshow (HL) test. An insignificant HL test statistic implies good calibration. In addition, the prediction model was subjected to 1,000 bootstrap resamples for internal validation to assess their predictive accuracies.

Statistical analysis was performed with SPSS Statistics for Windows, version 20.0 (IBM Corp, Armonk, NY, United States). The graphics of the nomogram and calibration curve were performed with R 4.0.4 (The R Foundation for Statistical Computing, Vienna, Austria) with the rms statistical packages.

## Results

**FIGURE 1:**
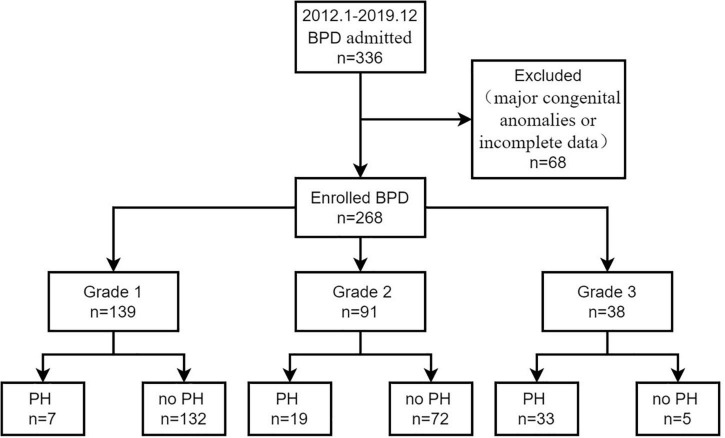
A flow diagram of study participants. BPD, bronchopulmonary dysplasia, PH, pulmonary hypertension.

During the study period, 336 preterm infants were diagnosed with BPD based on the 2019 criteria. In this study, 68 cases were excluded because of major congenital anomalies or incomplete data. A total of 268 preterm infants with BPD were enrolled, with 59 (22.0%) infants who developed BPD-PH beyond 36 weeks’ PMA in the BPD-PH group and 209 (78%) infants without PH in the no-PH group (Figure 1). The median age at admission was 21 days of life in the BPD-PH group and 4 days of life in the no-PH group. The incidences of PH in grade 1, grade 2, and grade 3 BPD infants were 5.0% (7/139), 20.9% (19/91), and 86.8% (33/38), respectively.

1. Univariate analyses of maternal and neonatal characteristics between the two groups

The characteristics and related risk factors were compared between the two groups (Table 1). Infants from the BPD-PH group were born at a lower gestational age with smaller birth weights than the infants from the no-PH group (*p* < 0.05). The incidences of pregnancy-induced hypertension (PIH), grade 3 BPD, early PH, SGA, RDS with surfactant use, pneumonia, severe IVH, and PDA ligation were significantly higher in the BPD-PH group (*p* < 0.05). The duration of hsPDA and IMV before 36 weeks’ PMA was much longer in infants with BPD-PH than in those without PH (*p* < 0.05).

**TABLE 1:** Maternal and neonatal characteristics between two groups.

Variables	BPD-PH (*n* = 59)	no-PH (*n* = 209)	*P*
Gestational age (weeks), mean (SD)	27.7 ± 1.7	28.4 ± 1.6	0.009*
Birth weight (g), mean (SD)	970 ± 224	1150 ± 250	0.000*
Male gender, *n* (%)	36 (61.0)	141 (67.5)	0.355**
SGA, *n* (%)	10 (16.9)	10 (4.8)	0.004**
Apgar score at 5 min, median (IQR)	8 (6, 9)	8 (6, 9)	0.350***
Delivery by C-section, *n* (%)	28 (47.5)	85 (40.7)	0.373**
Oligohydramnios, *n* (%)	4 (6.8)	17 (8.1)	0.732**
PROM > 24 h, *n* (%)	17 (28.8)	49 (23.4)	0.397**
pregnancy-induced hypertension, *n* (%)	12 (20.3)	16 (7.7)	0.008**
Antenatal corticosteroids, *n* (%)	13 (22.0)	75 (35.9)	0.059**
**BPD, *n* (%)**			
Grade 1	7 (5.0)	132 (95.0)	0.000**
Grade 2	19 (20.9)	72 (79.1)	
Grade 3	33 (86.8)	5 (13.2)	
RDS and surfactant use, *n* (%)	53 (89.8)	154 (73.7)	0.008**
Early PH, *n* (%)	43 (72.9)	63 (30.1)	0.000**
**Duration of hsPDA, *n* (%)**			
< 7 days	8 (13.6)	96 (45.9)	0.000**
7-13 days	2 (3.4)	21 (10.0)	
14-27 days	4 (6.8)	31 (14.8)	
≥ 28 days	45 (76.3)	61 (29.2)	
PDA ligation, *n* (%)	26 (44.1)	34 (16.3)	0.000**
pneumonia, *n* (%)	54 (91.5)	135 (64.6)	0.000**
NEC (II–III), *n* (%)	6 (10.2)	11 (5.3)	0.222**
Culture proven sepsis, *n* (%)	21 (35.6)	62 (29.7)	0.426**
IVH (III–IV), *n* (%)	8 (13.6)	10 (4.8)	0.034**
Duration of IMV before 36 weeks PMA (day), median (IQR)	25 (9,42)	7 (1,18)	0.000***

SD, standard deviation; SGA, small for gestational age; IQR, interquartile range; PROM, premature rupture of membrane; BPD, bronchopulmonary dysplasia; RDS, neonatal respiratory distress syndrome; PH, pulmonary hypertension; hsPDA, hemodynamically significant patent ductus arteriosus; NEC, necrotizing enterocolitis; IVH, intraventricular hemorrhage; IMV, invasive mechanical ventilation. *The *t*-test for metric variable if data are normally distributed; **the Chi-square test (big sample size) and Fisher’s exact test (small sample size) for categorical variables; ***the Mann–Whitney *U*-test for metric variables if data are not normally distributed.

2. Selected factors for the prediction model of BPD-PH

After univariable analysis, the variables of GA, BW, SGA, PIH, BPD, RDS with surfactant use, early PH, severe IVH, the duration of hsPDA, PDA ligation, pneumonia, and the duration of IMV before 36 weeks’ PMA were entered into the multivariate logistic regression analysis.

Multivariate logistic regression analysis showed that the independent predictive factors of BPD-PH were SGA, early PH, moderate to severe BPD (Grade 2 and 3 BPD), and the duration of hsPDA ≥ 28 days (Table 2).

**TABLE 2:** The multivariate logistic regression analysis to estimate risk for BPD-PH.

Variables	β	OR	95.0% CI	*P*
Constant	−4.617	0.010		
SGA	1.594	4.924	1.007–24.074	0.013
Early PH	1.205	3.337	1.358–8.202	0.009
BPD				
Grade 2	1.201	3.325	1.237–8.935	0.017
Grade 3	4.792	120.533	29.266–496.421	0.000
Duration of hsPDA ≥ 28 days	2.068	7.911	2.898–21.593	0.000

SGA, small for gestational age, PH, pulmonary hypertension, BPD, bronchopulmonary dysplasia, hsPDA, hemodynamically significant patent ductus arteriosus.

3. The predictive nomogram for the probability of BPD-PH

**FIGURE 2:**
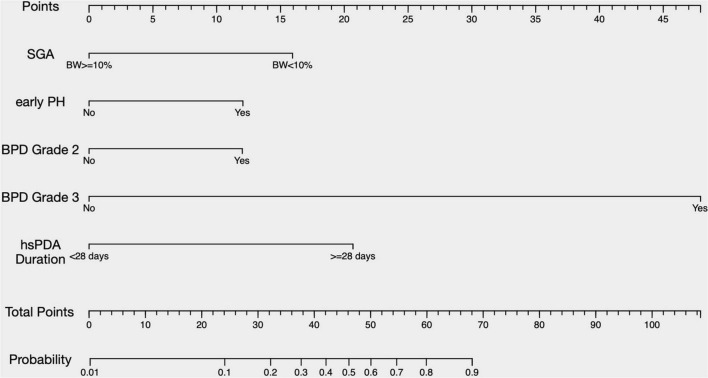
A nomogram predicting the risk of BPD-PH. The value of each of variable was given a score on the point scale axis. A total score could be easily calculated by adding each single score and, by projecting the total score to the lower total point scale, we were able to estimate the probability of BPD-PH. SGA, small for gestational age; PH, pulmonary hypertension; BPD, bronchopulmonary dysplasia; hsPDA, hemodynamically significant patent ductus arteriosus.

On the basis of the final regression analysis, a prediction model was established that incorporated the β coefficients of the predictors,


logit⁢(p)=⁢ln⁡p1-p=-4.617+1.594×SGA+1.205×Early⁢PH+1.201×Grade⁢ 2⁢BPD+4.792×Grade⁢ 3⁢BPD+2.068×duration⁢of⁢hsPDA

where, *p* represented the probability of developing BPD-PH. Other values of the predictors are as follows, SGA (no = 0, yes = 1), early PH (no = 0, yes = 1), Grade 2 BPD (no = 0, yes = 1), Grade 3 BPD (no = 0, yes = 1), and the duration of hsPDA (< 28 days = 0, ≥ 28 days = 1). A nomogram was developed based on the prediction model to improve the convenience of the model in clinical practice (Figure 2).

**FIGURE 3:**
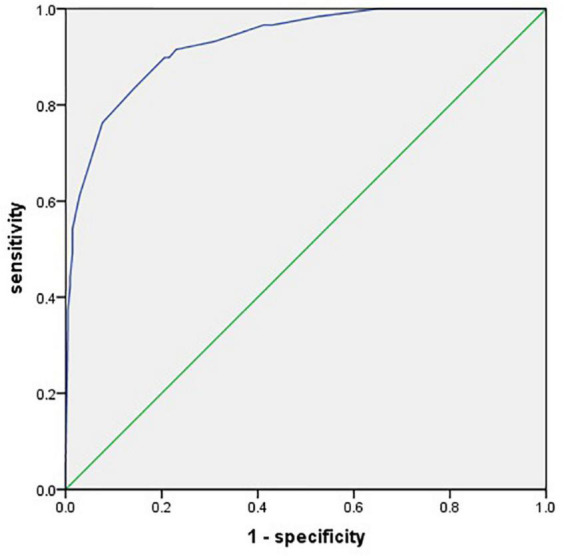
A receiver operating characteristic (ROC) curve for evaluating the prediction model’s discrimination performance [area under the ROC curve (AUC) = 0.930].

**FIGURE 4:**
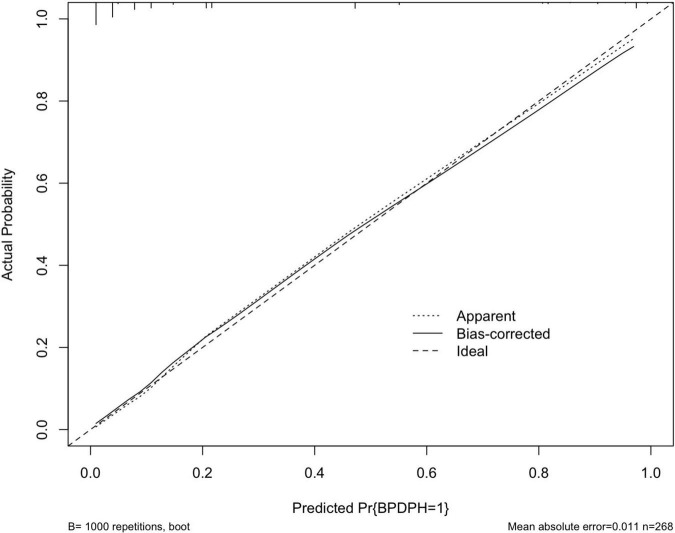
A calibration curve of the prediction model. The *x*- and *y*-axes represent the predicted risk and actual outcome, respectively. The dashed line indicates perfect prediction by an ideal model. The solid line depicts the model’s performance.

The area under the ROC curve (AUC) of the prediction model was 0.930 (95% CI, 0.895–0.965) (Figure 3). The Hosmer–Lemeshow test revealed no statistical significance (*p* = 0.976), suggesting a good fitting of the model. The calibration curve of the prediction model (Figure 4) demonstrated good calibration. The cutoff probability in this model is 0.173, with a sensitivity of 89.8% and a specificity of 79.4%, respectively.

## Discussion

Since PH is serious comorbidity of BPD with high mortality (31) and a lack of evidence-based therapies, it is of great importance to identify the risk factors and start early interventions to improve the long-term outcome. In this study, we established and assessed a prediction model for individually predicting patients with BPD who are likely to develop BPD-PH. The prediction model incorporated demographic and clinical characteristics and showed good discrimination and calibration performance. We further developed a nomogram, thus making it a convenient and valuable tool for clinical practice.

Several published studies have examined a wide variety of risk factors for the development of pulmonary hypertension in infants with BPD (6, 8, 21). Nagiub et al. (22) performed a meta-analysis to further analyze the effect size of risk factors for BPD-PH and showed that the duration of mechanical ventilation, prolonged NICU stay, oligohydramnios, the use of high frequency ventilation, SGA, sepsis, and severity of BPD were significant risk factors; while birth weight and gestational age were inversely related.

However, it is difficult to quantify the risk of developing BPD-PH according to one single predictive factor due to its low sensitivity and specificity. More importantly, PH that complicates BPD is frequently multifactorial, including perinatal stress, abnormal lung development, and postnatal diseases. Thus, how to transform these risk factors into a risk-based scoring system could help develop an evidence-based screening strategy for infants at high risk.

Prediction models by integrating multiple predictive factors have been used frequently in other conditions, whereas, there are few studies about prediction models for developing PH in infants with BPD. In the study by Trittmann et al. (23), they created a predictive model by combining both clinical and genetic data to predict the development of PH in patients with BPD. An ROC analysis showed the AUC of the prediction model of clinical data only, clinical and genetic data combined were 0.65 and 0.73, respectively. Due to the small cohort size of 20 cases of BPD-PH and 59 cases of BPD, the application of their model is limited and further validation in a larger independent prospective BPD cohort is needed. Compared with this study, our prediction model has relatively higher discrimination, with an AUC of 0.930.

In our prediction model, grade 3 BPD is the greatest contributor to the risk of BPD-PH, followed by the duration of hsPDA exposure, SGA, and early PH. The impact of the severity of BPD on BPD-PH was variable in previous studies. A few studies could not find an association between PH and the severity of BPD (24). In contrast, Arjaans et al. (25) revealed that infants with severe BPD were most at risk of developing PH, with a relative risk (*RR*) doubled (*RR* 2.7, 95% CI 1.7, 4.2). Our study reconfirms severe BPD as a strong risk factor for the development of PH, compared with mild or moderate BPD.

As a provincial referral center, all the cases were transferred from the maternal hospital or local hospital. Prolonged mechanical ventilation requirement and hsPDA unresponsive to pharmacologic therapy were common reasons for transport. The longer duration of hsPDA exposure is associated with the increasing risk of BPD in extremely preterm infants (26), which elucidates that hsPDA may delay lung disease recovery. Whether continued hsPDA exposure in infants with severe BPD may exacerbate BPD-PH is uncertain. Some studies reported no relationship between hsPDA and the development of BPD-PH (8, 9). However, in these reports, hsPDA was considered a categorical variable (present or absent) with no consideration of its duration. In our study, we found infants in the BPD-PH group were transferred to our unit much later than infants in the no-PH group, which means infants with BPD-PH had longer exposure to hsPDA. This also explained why univariable analysis showed the PDA ligation rate was much higher in the BPD-PH group than in the no-PH group because 76.3% of infants in the BPD-PH group had hsPDA exposure for ≥ 28 days.

Small for gestational age (SGA) has been identified to be a significant risk factor across some studies. Check et al. (21) further evaluated the associations between birth weight percentile and pulmonary hypertension at 36 weeks PMA in infants with moderate or severe BPD. In this study, infants with birth weights below the 25th percentile cutoff were at a higher risk of developing BPD-PH.

An early injury to the developing lung can impair angiogenesis and alveolarization, which results in the simplification of distal lung airspace and the clinical manifestations of BPD and PH. Previous prospective and retrospective studies in preterm infants have revealed that early PH is strongly associated with a high risk for the subsequent development of BPD at 36 weeks PMA (7, 27), but the results were conflicting on the association between early PH and the development of late PH (15, 28). However, evidence about the relationship between early PH and late BPD-PH is limited. In our cohort, early PH within 14 days of life is identified to be the independent risk factor of BPD-PH, which might improve the earlier detection and management of patients with BPD-PH.

Antenatal corticosteroids are well-known interventions that decrease the severity of RDS; however, the recent meta-analysis of the Cochrane Database shows that it is unclear if antenatal corticosteroids have any effect on the risk of BPD compared with placebo or no treatment (29). Neither of the two meta-analyses by Nagiub et al. (22) and Arjaans et al. (25) showed any effect of antenatal corticosteroids on BPD-PH. In our study, though infants with BPD-PH had received less antenatal corticosteroids, the *p*-value was above 0.05, we did not apply antenatal corticosteroids to the multivariate logistic regression analysis.

Presentations of BPD-PH are usually non-specific and easy to be neglected. Hence, active screening and treatment are recommended to minimize morbidity and mortality. Consensus recommendations for PH screening in infants with BPD have been developed by the Pediatric Pulmonary Hypertension Network (PPHNet), a multidisciplinary panel of PH experts (30). However, with the prediction model, it is possible to estimate the risk of BPD-PH precisely and optimize the utilization of medical resources. For patients with high risk, early and frequent screenings for PH may be considered individually. In addition, risk factors should be controlled more actively, such as the prompt treatment of hsPDA and optimization of respiratory support for those infants.

There are several limitations to our study. First, there might be selection bias in this retrospective study because we excluded infants without complete data or died before 36 weeks’ PMA. Echocardiograms were performed to evaluate the hemodynamic status of PDA every 2 or 3 days for very premature infants until PDA became hemodynamically insignificant. HsPDA exposure is a continuous variable, but we had to divide the cohort into four subgroups because of our inability to precisely calculate the duration of hsPDA (days). Second, only internal validation was performed because of the single-center study, in which the differences in epidemiology and clinical behavior that exist between different centers were not considered. Furthermore, genomic characteristics were not considered in our study. Genetic data combining clinical characteristics may improve the prediction model performance.

In conclusion, the prediction model based on the four risk factors predicts the development of BPD-PH with high sensitivity and specificity. External validation through multicenter prospective studies is necessary to further assess the generalizability of the prediction model.

## Data availability statement

The original contributions presented in this study are included in the article/supplementary material, further inquiries can be directed to the corresponding author.

## Author contributions

CW had primary responsibility for protocol development, preliminary data analysis, and writing of the manuscript. CW and YX participated in patient screening, enrollment, and data collection. ZC and LS participated in the design of the protocol and data analyses. XM supervised the design and execution of the study and contributed to the revision of the manuscript. LD supervised the design and performed the final data analyses. All authors contributed to the article and approved the submitted version.

## Conflict of interest

The authors declare that the research was conducted in the absence of any commercial or financial relationships that could be construed as a potential conflict of interest.

## Publisher’s note

All claims expressed in this article are solely those of the authors and do not necessarily represent those of their affiliated organizations, or those of the publisher, the editors and the reviewers. Any product that may be evaluated in this article, or claim that may be made by its manufacturer, is not guaranteed or endorsed by the publisher.
